# Habitat-based radiomics analysis for evaluating immediate response in colorectal cancer lung metastases treated by radiofrequency ablation

**DOI:** 10.1186/s40644-024-00692-w

**Published:** 2024-03-26

**Authors:** Haozhe Huang, Hong Chen, Dezhong Zheng, Chao Chen, Ying Wang, Lichao Xu, Yaohui Wang, Xinhong He, Yuanyuan Yang, Wentao Li

**Affiliations:** 1 Department of Interventional Radiology, Fudan University Shanghai Cancer Center, 270 Dongan Road, Xuhui District, Shanghai, 200032 China; 2 Department of Oncology, Shanghai Medical College, Fudan University, Xuhui District, 130 Dongan Road, Shanghai, 200032 China; 3 Department of Medical Imaging, Shanghai Mental Health Center, Shanghai Jiao Tong University School of Medicine, 600 South Wanping Road, Xuhui District, Shanghai, 200030 China; 4 Laboratory for Medical Imaging Informatics, Shanghai Institute of Technical Physics, Chinese Academy of Science, 500 Yutian Road, Hongkou District, Shanghai, 200083 China; 5 University of Chinese Academy of Sciences, 19 Yuquan Road, Shijingshan District, Beijing, 100049 China

**Keywords:** Colorectal neoplasms, Lung metastasis, Radiofrequency ablation, Radiomics, Habitat imaging, Peritumoral micro-environment, Immediate response

## Abstract

**Purpose:**

To create radiomics signatures based on habitat to assess the instant response in lung metastases of colorectal cancer (CRC) after radiofrequency ablation (RFA).

**Methods:**

Between August 2016 and June 2019, we retrospectively included 515 lung metastases in 233 CRC patients who received RFA (412 in the training group and 103 in the test group). Multivariable analysis was performed to identify independent risk factors for developing the clinical model. Tumor and ablation regions of interest (ROI) were split into three spatial habitats through K-means clustering and dilated with 5 mm and 10 mm thicknesses. Radiomics signatures of intratumor, peritumor, and habitat were developed using the features extracted from intraoperative CT data. The performance of these signatures was primarily evaluated using the area under the receiver operating characteristics curve (AUC) via the DeLong test, calibration curves through the Hosmer-Lemeshow test, and decision curve analysis.

**Results:**

A total of 412 out of 515 metastases (80%) achieved complete response. Four clinical variables (cancer antigen 19–9, simultaneous systemic treatment, site of lung metastases, and electrode type) were utilized to construct the clinical model. The Habitat signature was combined with the Peri-5 signature, which achieved a higher AUC than the Peri-10 signature in the test set (0.825 vs. 0.816). The Habitat+Peri-5 signature notably surpassed the clinical and intratumor radiomics signatures (AUC: 0.870 in the test set; both, *p* < 0.05), displaying improved calibration and clinical practicality.

**Conclusions:**

The habitat-based radiomics signature can offer precise predictions and valuable assistance to physicians in developing personalized treatment strategies.

**Supplementary Information:**

The online version contains supplementary material available at 10.1186/s40644-024-00692-w.

## Introduction

The lung ranks as the second most frequent location for metastases from colorectal cancer (CRC) [1]. Despite the importance of surgery, published reports indicate varying 5-year survival rates of 24 to 56% following surgical removal of CRC lung metastases [2]. European Society for Medical Oncology (ESMO) guidelines for metastatic CRC suggest considering local ablation alongside resection, based on factors like tumor size, number, location, lung tissue loss, comorbidity, and other relevant considerations [3].

Computed tomography (CT)-guided percutaneous radiofrequency ablation (RFA) stands as a viable choice for treating small lung metastases, especially those under 3 cm [4, 5]. However, due to the lack of histological evidence confirming complete ablation and a reported recurrence rate of 32.6% [6], assessing ablation outcomes after RFA remains challenging. The usual assessment of ground-glass opacity (GGO) [7], linked to residual tumor and recurrence [8], can be impacted by intraoperative intra-alveolar hemorrhage (IAH) or atelectasis [4, 9, 10]. The initial 3-month inflammatory response around the ablated lesion complicates early efficacy evaluation [11]. Hence, there’s a need to objectively define and evaluate the immediate ablation outcomes for CRC lung metastases.

In the context of personalized and precise treatments, radiomics can extract high-dimensional quantitative features from medical images. These features encompass data about tumor heterogeneity and the microenvironment [12, 13], enabling a more accurate assessment of traits and treatment response [14, 15]. Peritumoral features have been proposed to enhance radiomic models’ predictive abilities [16–19]. Unlike previous methods, a novel approach divides tumors into subregions known as habitats, containing voxels with similar attributes and consistent tumor biology [20]. This approach improved intratumoral heterogeneity quantification [21, 22].

To the best of our knowledge, no studies have aimed to develop habitat-based radiomics analysis to predict early RFA efficacy in CRC lung metastases. In this study, we employed a new radiomics approach to identify imaging biomarkers within intratumoral, peritumoral, and sub-regional zones. This enables assessing the immediate response to RFA in CRC lung metastases.

## Methods

### Patient selection and clinicopathological information

**Fig. 1:**
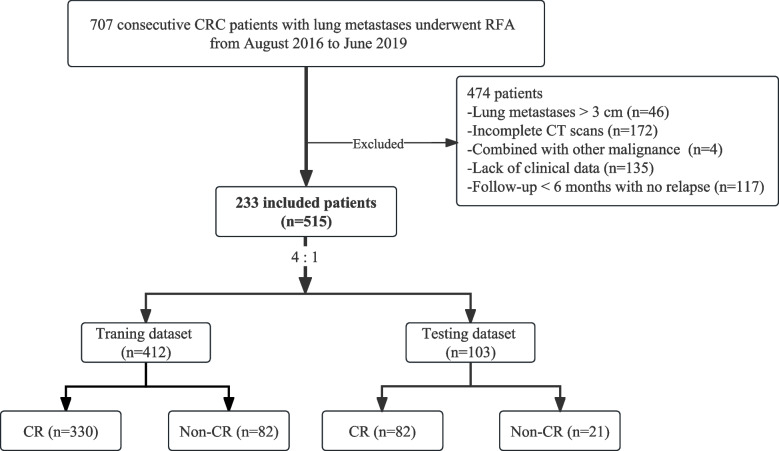
Flow diagram of the enrolment patients

Due to the retrospective nature of this study, patient informed consent was waived. We included 233 consecutive CRC patients with lung metastases who underwent initial RFA between August 2016 and June 2019. Inclusion criteria were: (1) confirmed CRC through histology; (2) lung metastases treated with RFA, ≤ 3 cm in maximum diameter; (3) comprehensive medical records with clinical variables and CT data from procedure and follow-up; (4) technically successful ablation; (5) adequate normal organ function. Exclusion criteria were: (1) receiving other local treatments like radiotherapy or re-ablation; (2) inability to tolerate RFA; (3) concurrent malignant tumors or extrapulmonary metastasis. Patients with multiple nodules were included, analyzing each nodule individually [10, 23, 24]. A cohort of 515 lung metastases in 233 CRC patients who underwent RFA was enrolled and randomly divided into training and test cohorts at a 4:1 ratio (Fig. 1). Supplementary data 1 provided detailed RFA equipment and procedure information.

The clinical variables including age, gender, serum tumor markers (carcinoembryonic antigen (CEA) and cancer antigen 19–9 (CA19–9)), lymphadenopathy at diagnosis, concomitant systemic treatment, and primary tumor location were collected within 1 week before RFA. Radiological data from intraoperative CT scans included pulmonary metastases size, location, proximity to vital structures like the heart or major blood vessels (> 3 mm in diameter), distance to the pleura or diaphragm (within 1 cm), electrode type, and complications such as IAH or pneumothorax.

### CT examination protocol and local efficacy assessment

Pre- and immediate post-ablation CT scans were conducted using the United Imaging uCT 760 (United Imaging Medical Technology Inc.) and Philips Brilliance 64 slice (Philips Medical Systems Inc.) machines. Settings were: 200 mA, 120 kVp, 0.5 s/round, with 1 mm or 1.5 mm section thickness. The images were reconstructed using iterative reconstruction, and the resulting CT data was stored in the. DICOM format.

A contrast-enhanced chest CT was conducted as the baseline 1 month after ablation [25], followed by additional scans every 2 to 3 months. The treatment’s local efficacy was assessed by two experienced radiologists who were unaware of clinical data. Evaluation was grounded in chest-enhanced CT scans performed at least 6 months after RFA, adhering to the modified response evaluation criteria in solid tumors (mRECIST) criteria [11, 26]. Should there be differences in interpretation between the radiologists, consultation with a senior expert boasting over 20 years of experience was pursued. Complete response (CR) was determined by the presence of cavity, fibrosis, or nodule without enhancement. In contrast, the presence of irregular nodular, scattered, or eccentric patterns of peripheral enhancement within 1 cm of the ablation area in two consecutive CT scans denoted a non-complete response (non-CR).

### Workflow of radiomics analysis

**Fig. 2:**
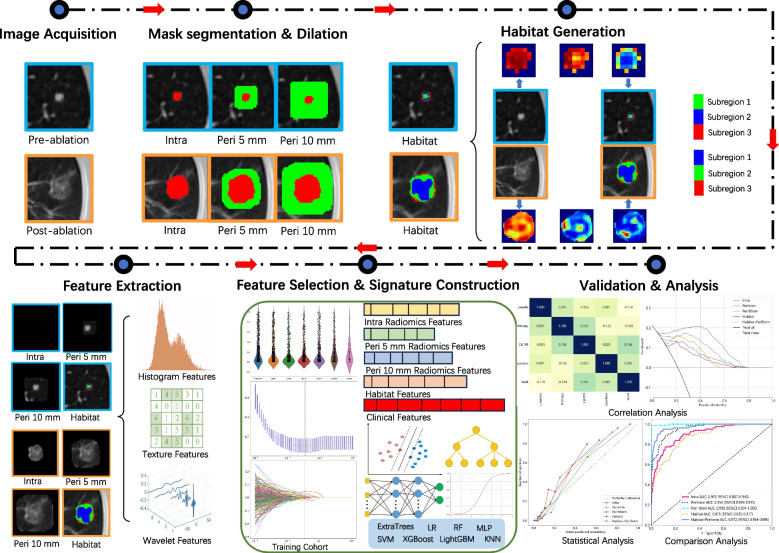
Workflow of radiomics analysis

The radiomics analysis was executed through a series of steps: image segmentation, feature extraction, feature selection, signature construction, and evaluation (Fig. 2).

### Advanced image processing and mask segmentation

To enhance the robustness of medical image analysis, preprocessing techniques were applied. The CT images were uniformed to a common resolution of 1 mm × 1 mm × 1 mm by the B-spline interpolation algorithm, and then the window width was adjusted within the range of - 1200 Hu to 600 Hu and the intensity was scaled within the range of 0 ~ 255.

Recently, numerous studies have demonstrated that the improved methods based on U-Net performed well in the segmentation of pulmonary nodules, which were trained on the Image Database Resource Initiative (IDRI) created by the US Institutes of Health based on the Lung Image Database Consortium (LIDC) [27–31]. We also have trained a 3D UNet model based on the open-source dataset to segment the target lesions and immediate ablation regions from pre- and postoperative CT images, with a Dice coefficient of 83.04% [32]. These masks were subsequently verified by two junior radiologists (HZH and HC, 8 years of specialized chest imaging) and the necessary adjustments have been made to guarantee accuracy and repeatability using the ITK-SNAP (version 3.8.0, http://www.itksnap.org). If they had disagreements, it would be determined in consultation with the senior expert (WTL, 30 years of specialized chest imaging).

### Peritumoral region dilation

The regions of interest (ROI) were expanded using the mask padding toolkit provided by the Onekey AI platform. We evaluated the impact of different peritumoral sizes on model predictability by applying dilation intervals of 5 mm. Any ROIs extending beyond the lungs or overlapping with the heart, major blood vessels, or diaphragm were manually adjusted.

### Habitat generation

Local features, including local entropy and energy values, were extracted from each voxel within VOI. These feature vectors represented diverse aspects of voxel properties. A 77-dimensional feature vector (Supplementary data 2) was generated for each block using a 3 × 3 × 3 non-overlapping moving window. The Calinski-Harabasz (CH) value selection method [33] was used to determine the optimal number of clusters. Subsequently, the K-means method was employed to cluster sub-regions for each sample.

### Feature extraction and selection

Handcrafted features extracted using the Pyradiomics tool (version 3.0.1) were categorized into three groups: geometry, intensity, and texture (Supplementary data 2), following the guidelines of the imaging biomarker standardization initiative (IBSI). Unsupervised clustering yielded varying physical meanings for the habitat extracted from the subregions with identical centers. To mitigate this, mean feature values were computed.

Robustness was assessed through test-retest and inter-rater analyses, with the intraclass correlation coefficient (ICC) set at a threshold of ≥0.85. Nonetheless, ICC was not suitable for assessing the unsupervised habitat signature. All features were standardized using Z-scores to maintain a normal distribution, followed by a t-test to retain radiomic features with *p*-value < 0.05. Pearson’s correlation coefficient was computed to pinpoint highly consistent features with a coefficient > 0.9. The greedy recursive deletion strategy was then employed to filter out the highly redundant features. To curb overfitting, the minimum redundancy maximum relevance (mRMR) algorithm selected the top 8 features for each modality. The final features set was determined using the least absolute shrinkage and selection operator (LASSO) regression. LASSO adjusted parameter λ to assign zero regression coefficients to irrelevant features. Optimal λ value selection involved 10-fold cross-validation with minimum criteria, aiming for the lowest mean square error (MSE).

### Signature construction

Several radiomics signatures were formulated based on distinct regions: intratumor and ablated area (Intra), intratumor with expanded tumor and ablated regions (5 mm and 10 mm, Peri-X), intratumor and ablated area subregions (Habitat), and intratumor combined with peritumoral regions. Additionally, the optimal peritumoral region was integrated with the tumor microenvironment habitat, termed Habitat + Peri-X. The Clinical signature was created from independent risk factors identified via multivariate logistic analysis. Commonly used machine learning models, including logistic regression (LR), support vector machine (SVM), K-nearest neighbor (KNN), random forest (RF), extremely randomized trees (ExtRa Trees), eXtreme gradient boosting (XGBoost), light gradient boosting machine (LightGBM), and multi-layer perceptron (MLP), were employed for model construction. Optimal hyperparameters for each model were determined using five-fold cross-validation and the Grid-search algorithm.

### Performance evaluation

The performance of various signatures was verified using an independent test dataset, generating receiver operating characteristic (ROC) curves to calculate the corresponding area under the curve (AUC). The Delong test was used to compare predictive performance differences among the models [34]. Additionally, accuracy, sensitivity, specificity, positive predictive value (PPV), and negative predictive value (NPV) were computed. The Youden index determined the optimal cut-off value maximizing the sum of sensitivity and specificity [35]. Calibration curves were plotted to assess calibration accuracy, alongside the Hosmer-Lemeshow (HL) test [36] (A significant test statistic implies that the model does not calibrate perfectly.). Moreover, decision curve analysis (DCA) gauged the clinical utility of predictive signatures [37].

### Statistical analysis

Statistical analyses were performed using IBM SPSS (version 26.0). Continuous variables were presented as mean ± standard deviation (SD) and compared using the Man-Whitney U test. Categorical variables were expressed as counts with percentages and compared using the Chi-square or Fisher test. Variables with a *P*-value < 0.05 in univariate regression analysis were included in multivariable analysis. Variables with a P-value < 0.05 in multivariable analysis were considered independent predictors linked to immediate response. All statistical tests were two-sided with a significance level set at *P* < 0.05.

## Results

### Baseline characteristics

The training dataset consisted of 412 lesions (330 CR and 82 non-CR) selected via random division, while 103 lesions (82 CR and 21 non-CR) contributed the independent test dataset (Table 1).

**Table 1:** Characteristics of patients and CRC lung metastases

Characteristics	Training dataset			Test dataset			*P* value (N_1_ vs N_2_)
	N_1_ = 412	CR = 330	Non-CR = 82	N_2_ = 103	CR = 82	Non-CR = 21	
Age (years)^#^	57.91 ± 10.42	57.74 ± 10.37	58.41 ± 10.69	57.48 ± 10.89	58.67 ± 10.81	52.80 ± 9.85	0.59
Gender							0.68
Male	234	184	50	61	48	13	
Female	178	146	32	42	34	8	
Tumor markers							
CEA (ng/ml)^#^	14.89 ± 44.12	12.48 ± 37.91	27.13 ± 65.92	13.49 ± 20.80	11.50 ± 16.80	20.84 ± 30.89	0.71
CA19–9 (U/ml)^#^	29.73 ± 56.78	25.41 ± 48.32	51.75 ± 88.38	20.67 ± 30.69	18.91 ± 29.86	27.01 ± 32.95	0.29
Lymphadenopathy at diagnosis							0.03
Yes	282	218	64	61	50	11	
No	130	112	18	42	32	10	
Concomitant systemic treatment							0.10
Yes	276	231	45	59	47	12	
No	136	99	37	44	35	9	
Initial tumor location							0.60
Rectum	274	216	58	76	57	19	
Sigmoid—left colon	58	50	8	11	10	1	
Transverse—right colon	75	60	15	14	13	1	
Caecum	5	4	1	2	2	0	
Nodule size (mm)^#^	1.21 ± 0.66	1.12 ± 0.38	1.35 ± 0.42	1.27 ± 0.67	1.20 ± 0.48	1.38 ± 0.71	0.31
Lung metastases location							0.22
RUL	98	87	11	23	19	4	
RML	39	32	7	18	16	2	
RLL	78	57	21	16	8	8	
LUL	94	79	15	22	19	3	
LLL	103	75	28	24	20	4	
Distance 1							0.28
> 1 cm	340	277	63	79	62	17	
< 1 cm	72	53	19	24	20	4	
Distance 2							0.08
> 1 cm	161	131	30	49	39	10	
< 1 cm	251	199	52	54	43	11	
Electrode type							1.00
Expandable	380	310	70	94	76	18	
Straight	32	20	12	9	6	3	
Pneumothorax							1.00
Yes	115	93	22	28	22	6	
No	297	237	60	75	60	15	
IAH							0.01
Yes	108	93	15	40	34	6	
No	304	237	67	63	48	15	

*CRC* colorectal cancer, *CEA* carcinoembryonic antigen, *CA19–9* cancer antigen 19–9, *CR* complete response, *RUL* right upper lobe, *RML* right middle lobe, *RLL* right lower lobe, *LUL* left upper lobe, *LLL* left lower lobe, *Distance 1* the distance between the lesion and the great vessels or mediastinum, *Distance 2* the distance between the lesion and the pleura or diaphragm, *IAH* intra-alveolar hemorrhage

^**#**^ Mean ± SD

### Establishment of clinical models

Univariate logistic regression analysis (Table 2) revealed significant factors for identifying ablated lesions: CEA, CA19–9, concomitant systemic treatment, lung metastases location, and electrode type (*P* < 0.05). Multivariate regression analysis indicated that CA19–9 (odds ratio [OR] = 1.002, *P* < 0.001), concomitant systemic treatment (OR = 0.916, *P* = 0.042), lung metastases location (OR = 1.032, *P* = 0.019), and electrode type (OR = 0.778, *P* = 0.004) were independent factors influencing ablation effect, forming the basis for clinical models construction.

**Table 2:** Uni- and multivariate analysis of clinical and radiological characteristics

Characteristics	Univariate analysis		Multivariate analysis	
	OR (95%CI)	*P* value	OR (95%CI)	*P* value
Age	1.001(0.998–1.004)	0.628		
Gender	1.047(0.975–1.123)	0.285		
CEA	1.001(1.001–1.002)	0.005*	1.001(1.0–1.001)	0.131
CA 19–9	1.002(1.001–1.002)	< 0.001*	1.002(1.001–1.002)	**< 0.001#**
Lymphadenopathy at diagnosis	1.091(1.013–1.175)	0.052		
Concomitant systemic treatment	0.895(0.832–0.964)	**0.013**	0.916(0.853–0.983)	**0.042#**
Initial tumor location	0.982(0.941–1.026)	0.500		
Nodule size	0.956(0.608–1.307)	0.642		
Lung metastases location	1.033(1.010–1.015)	0.020*	1.032(1.009–1.005)	**0.019#**
Distance 1	1.092(0.995–1.198)	0.119		
Distance 2	1.006(0.935–1.081)	0.897		
Electrode type	0.753(0.652–0.869)	0.001*	0.778(0.675–0.897)	**0.004#**
Pneumothorax	1.012(0.934–1.096)	0.808		
IAH	0.933(0.860–1.013)	0.165		

*OR* odds ratio, *95%CI* 95% confidence interval, *CEA* carcinoembryonic antigen, *CA19–9* cancer antigen 19–9, *Distance 1* the distance between the lesion and great vessels or mediastinum, *Distance 2* the distance between the lesion and pleura or diaphragm, *IAH* intra-alveolar hemorrhage. The bold *p* values in the univariate (*) and multivariate analysis (#) mean < 0.05

### Sub-region cluster and feature selection

**Fig. 3:**
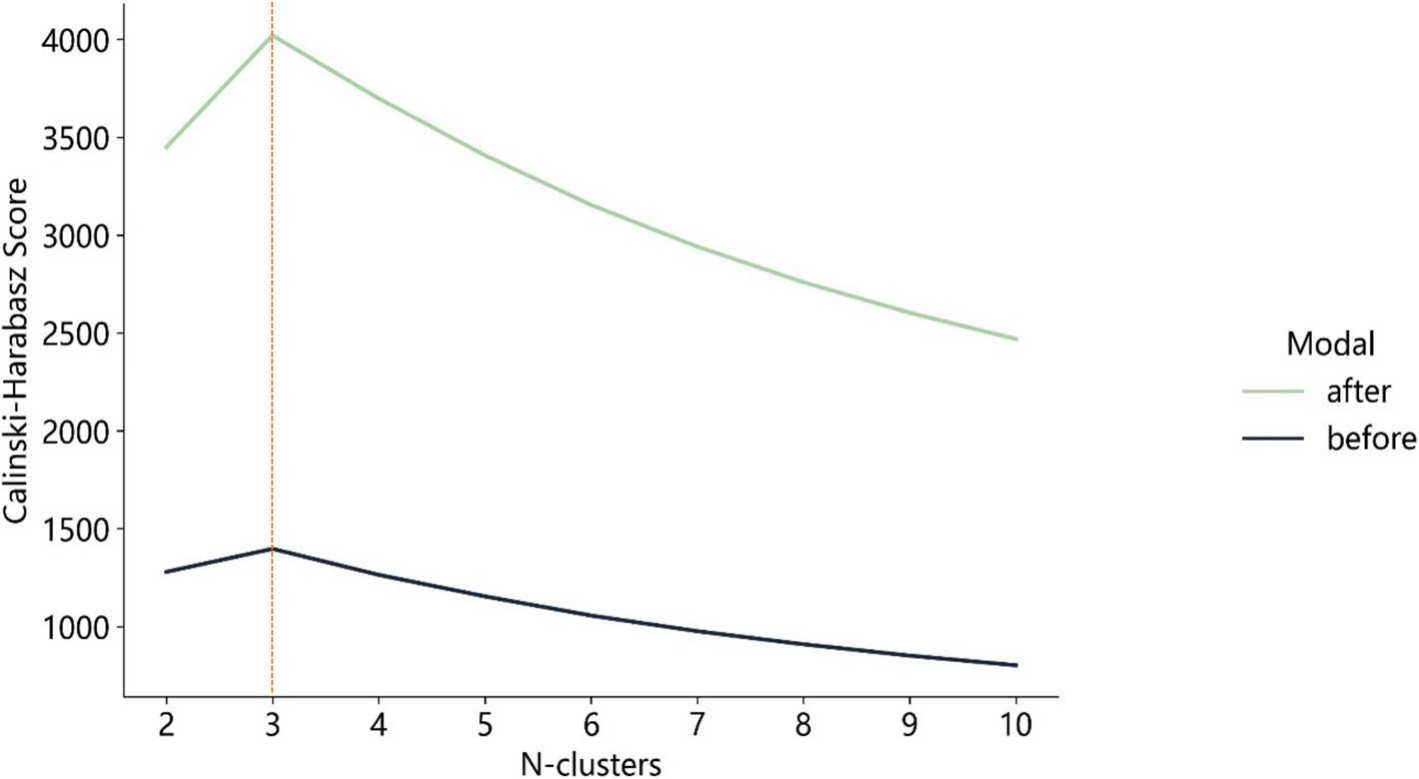
Calinski–Harabasz score plot. The red dotted line represented the optimal value beyond which the scores started to decrease in the radiomics features from CT images before (black line) and after (green line) ablation

Optimal CH value emerged when tumors were clustered into three sub-regions in the entire cohort (Fig. 3). To enhance the feature set, features from both pre- and post-ablation images were fused, resulting in 3668 features. Features were extracted via Pyradiomics (http://pyradiomics.readthedocs.io).

Radiomics features with non-zero coefficients were selected using the LASSO method with the best lambda (Supplementary data 3). For habitat-based radiomics signature, lambda of 0.0095 yielded the best, selecting 71 features. These comprised 20 habitat features (8 pre- and 12 post-RFA) and 51 peritumoral features (29 pre- and 22 post-RFA).

### Performance and comparison of signatures

Based on the analysis of prediction performance (Supplementary data 3), the Intra radiomics signature employed RF, the Peri-X radiomics signature utilized LightGBM, the Habitat radiomics signature was developed using ExtRa Trees, while the Habitat+Peri-5 radiomics and clinical signatures were constructed with XGBoost.

**Fig. 4:**
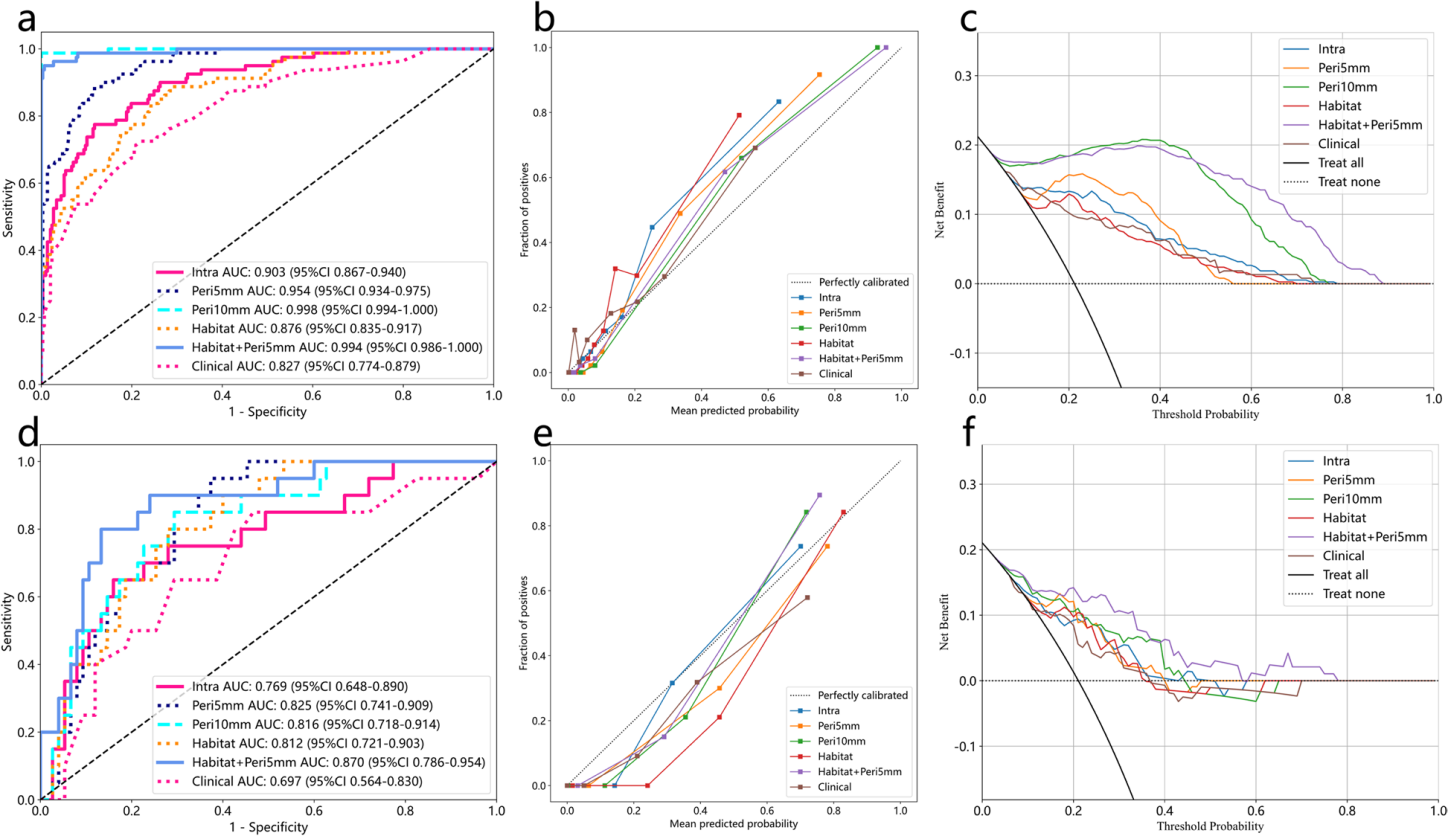
The receiver operating characteristic (ROC) curves, calibration curves, and decision curve analysis (DCA) of all signatures in the training cohort (**a, b, c**), and test cohort (**d, e, f**)

Summary of predictive performance for clinical and radiomics signatures was presented in Table 3 and Fig. 4. The clinical signature achieved AUC values of 0.827 (training) and 0.697 (test), hinting at potentially limited generalizability to unseen data. The Peri-5 signature outperformed Peri-10 in test AUC, prompting its combination with the Habitat signature. The Habitat+Peri-5 signature excelled in both training (AUC 0.972) and test (AUC 0.846) sets (Fig. 4 a and d). Moreover, calibration curves of the Habitat + Peri-5 signature exhibited robust concurrence between observed and projected probabilities (Fig. 4 b and e). The HL test yielded insignificant deviations (training cohort: *P* = 0.212; test cohort: *P* = 0.283), signifying conformity. DCA portrayed the enhanced clinical benefit of the fusion signature over other signatures (Fig. 4 c and f).

**Table 3:** Performance comparison of all signatures

Signature	ACC	AUC	95%CI	Sensitivity	Specificity	PPV	NPV	Youden	Cohort
Clinical	0.777	0.827	0.774–0.879	0.713	0.795	0.483	0.911	0.278	Training
Intra	0.859	0.903	0.867–0.940	0.775	0.882	0.639	0.936	0.252	Training
Peri-5	0.883	0.954	0.934–0.975	0.887	0.882	0.670	0.967	0.236	Training
Peri-10	0.997	0.998	0.995–1.000	0.988	1.000	1.000	0.997	0.375	Training
Habitat	0.748	0.876	0.835–0.917	0.887	0.710	0.452	0.959	0.201	Training
Habitat+Peri-5	0.984	0.994	0.986–1.000	0.950	0.993	0.974	0.987	0.366	Training
Clinical	0.600	0.697	0.564–0.830	0.850	0.541	0.327	0.930	0.193	Test
Intra	0.800	0.769	0.648–0.890	0.650	0.851	0.520	0.900	0.238	Test
Peri-5	0.695	0.825	0.741–0.909	0.950	0.635	0.404	0.979	0.189	Test
Peri-10	0.737	0.816	0.718–0.914	0.850	0.716	0.436	0.946	0.179	Test
Habitat	0.737	0.812	0.721–0.903	0.800	0.730	0.432	0.931	0.212	Test
**Habitat + Peri-5**	**0.853**	**0.870**	**0.786–0.954**	**0.800**	**0.867**	**0.615**	**0.942**	**0.274**	**Test**

*ACC* accuracy, *AUC* the area under the curve, *95%CI* 95% confidence interval, *PPV* positive predictive value, *NPV* negative predictive value. The bold values mean the best performance of the signature integrating habitat and radiomics features of peritumoral 5 mm

## Discussion

This study systematically explored the tumor microenvironment through a comprehensive evaluation of intra- and peritumoral regions in pre- and post-ablation CT scans. As a result, we devised predictive signatures for early ablation efficacy in CRC lung metastases treated with RFA, leveraging sub-regional radiomics features. These signatures provided effective tools for tailoring treatment strategies in CRC patients with lung metastases.

Radiomics analysis enables the extraction and characterization of a broad range of information reflecting underlying biological diversity in a cost-effective manner [12]. A radiomics signature, composed of multiple features, serves as a robust prognostic biomarker that could complement clinical data [38]. The overestimation of the completely necrotic region as GGO on CT [39] indicates the need to ablate peritumoral lung parenchyma within 5 to 10 mm for complete ablation [9, 40]. Previous studies have highlighted the utility of combining intra- and peritumoral radiomics features for improved treatment response prediction [16, 19]. These findings underscore the importance of peritumoral features [16–18, 41]. Therefore, our study aimed to explore the predictive performance of peritumoral radiomics features by assessing the impact of peritumoral region sizes. The results demonstrated that the radiomics signature derived from a 5 mm dilated distance outside both the tumor and ablated area yielded the highest prediction performance compared to the intra- and peritumoral 10 mm regions. This signature exhibited high AUC values and low over-fitting, emphasizing the significance of peritumoral features in radiomics and the influence of peritumoral size on prediction performance.

**Fig. 5:**
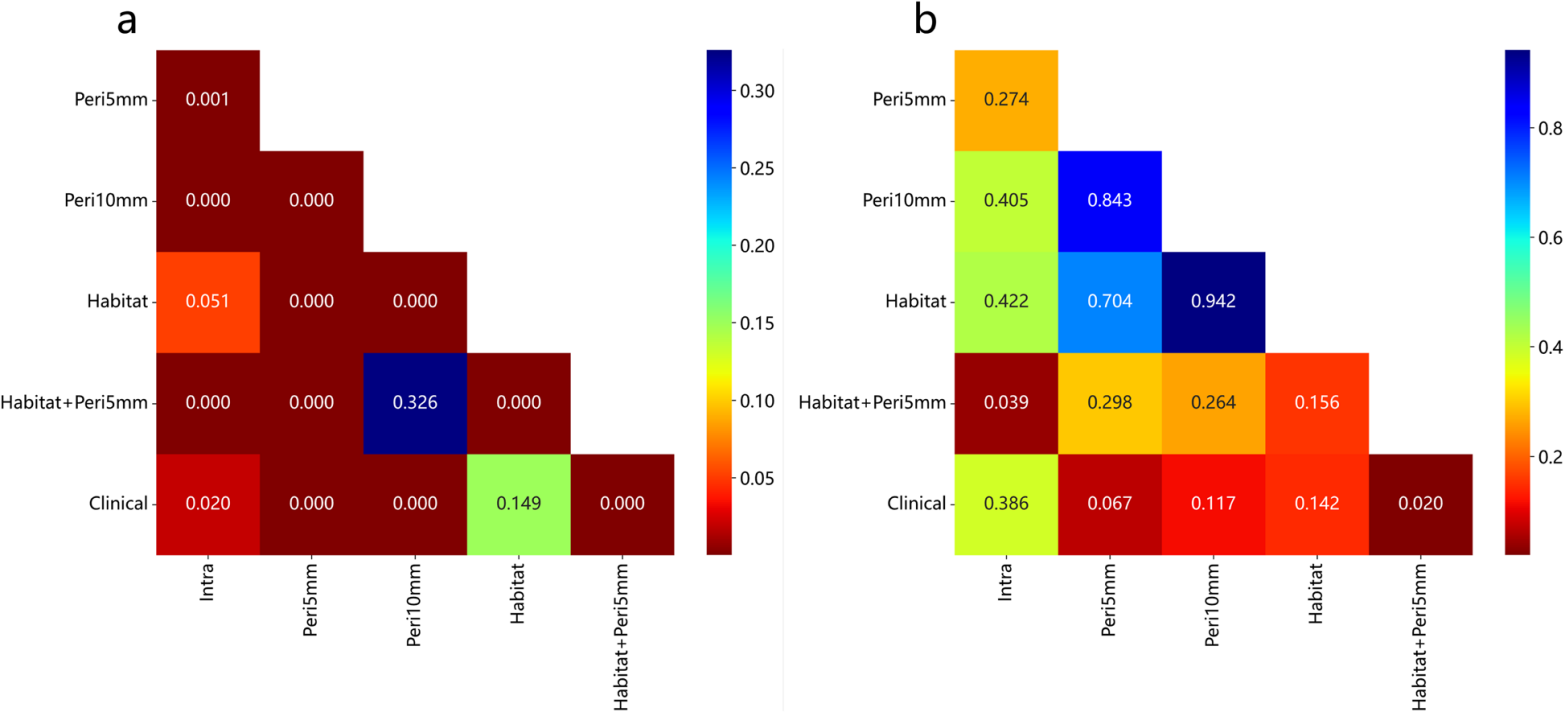
The results of the DeLong test in the training cohort (**a**), and test cohort (**b**)

The sub-region cluster analysis conducted in this study highlighted the importance of sub-region analysis in capturing CRC lung metastases’ tumor heterogeneity. By optimizing the number of clusters, we identified three spatially distinct habitats through K-means clustering. These habitats within the ablation area represented various characteristics: the inner region possibly indicated necrotic areas, the middle region potentially signified effusion areas, and the edge region likely represented congested areas. These findings were consistent with the histopathological results, demonstrating an inner-to-outer transition [39]. As anticipated, the habitat signature, derived from radiomics features within these unique subregions, contributed valuable information in assessing early effects after RFA. Furthermore, by combining habitat and peritumoral signatures, we achieved higher AUC values (training cohort: 0.972, test cohort: 0.870). This outcome validated the superiority of the Habitat+Peri-5 signature over the Intra radiomics signature (*P* = 0.039, Fig. 5).

Furthermore, multivariate regression analyses identified several clinical variables, including CA19–9 levels, lung metastases location, concomitant systemic treatment, and electrode type. Notably, lung metastases location, particularly in the lower lobes, emerged as an independent risk factor, possibly due to the influence of respiratory movements on accurate probe positioning. Conversely, concomitant systemic treatment and the use of expandable electrodes were protective factors against treatment failure, evident from OR values below 1. Importantly, the Habitat + Peri-5 signature demonstrated a significantly improved AUC value (*P* = 0.020, Fig. 5) compared to the clinical model (training cohort: 0.827, test cohort: 0.697), underscoring enhanced precision and clinical utility, as supported by the HL test and DCA results.

A study by Markich et al. [10] examined clinical, radiological, technical, and radiomics features examined before and after RFA to evaluate local control in 48 CRC patients with 119 lung metastases. However, their reliance on CT scans taken 48 hours post-ablation hindered real-time procedure assessment, delaying timely interventions. To address this limitation, our recent study [42] integrated relative radiomics features from pre- and immediate post-RFA CT scans with clinical and radiological variables from 479 lung metastases in 198 CRC patients. This aimed to establish a novel multimodal data fusion model for evaluating immediate RFA efficacy. Liu et al. [43] explored intratumor density heterogeneity changes following microwave ablation (MWA) of pulmonary tumors, utilizing radiomics-based CT features for prognostic value in predicting treatment response. Additionally, Zhu et al. [44] developed intra- and peritumoral radiomics models based on post-operative CT images to predict early MWA efficacy in malignant lung tumors, validating the predictive ability. However, neither study explored the optimal peritumoral region nor assessed the impact of peritumoral region sizes. Also, the inclusion of primary and metastatic lung tumors in both studies raised concerns about disease heterogeneity.

This study bears several limitations that warrant acknowledgment. Firstly, it was a single-center retrospective study with a limited sample size. Therefore, a more extensive, multi-center prospective study is essential to validate the generalizability of our signature and ascertain its utility in clinical settings. Secondly, the spatially distinct habitats identified through K-means clustering could not be pathologically confirmed due to technical challenges and ethical considerations. However, pursuing such a correlation might entail unnecessary surgeries.

## Conclusion

In summary, this study introduces a pioneering signature that combines habitat and peritumoral radiomics to access immediate response and predict outcomes of RFA in CRC patients with lung metastases. The habitat-based radiomics signature holds the potential for advancing precision medicine and shaping treatment strategies.

### Supplementary Information

**Supplementary material 1.**

## Data Availability

The datasets used and/or analyzed during the current study are available from the corresponding author on reasonable request.

## Abbreviations

AUC: Area under ROC curve
ACC: Accuracy
CA199: Cancer antigen 199
CEA: Carcinoembryonic antigen
CH: Calinski–Harabasz
CI: Confidence interval
CR: Complete response
CRC: Colorectal cancer
CT: Computed tomography
DCA: Decision curve analysis
ESMO: European Society for Medical Oncology
ExtRa: Trees Extremely randomized trees
GGO: Ground-glass opacity
HL: Hosmer-Lemeshow
IAH: Intra-alveolar hemorrhage
IBSI: Imaging biomarker standardization initiative
ICC: Intraclass correlation coefficient
IDRI: Image Database Resource Initiative
KNN: K-nearest neighbor
LASSO: Least absolute shrinkage and selection operator
LIDC: Lung Image Database Consortium
LightGBM: Light gradient boosting machine
LR: Logistic regression
MLP: Multi-layer perceptron
mRECIST: Modified response evaluation criteria in solid tumors
mRMR: Minimum redundancy maximum relevance
MSE: Mean square error
MWA: Microwave ablation
NPV: Negative predictive value
OR: Odds ratio
PPV: Positive predictive value
RF: Random forest
RFA: Radiofrequency ablation
ROC: Receiver operating characteristic
ROI: Region of interest
SD: Standard deviation
SVM: Support vector machine
VOI: Volumes of interest
XGBoost: eXtreme gradient boosting
