# Supplementary material for: Habitat-based radiomics analysis for evaluating immediate response in colorectal cancer lung metastases treated by radiofrequency ablation
Source: Cancer Imaging. 2024 Mar 26;24:44. doi: 10.1186/s40644-024-00692-w (PMC10964536; doi:10.1186/s40644-024-00692-w)
Supplement: Supplementary file 1 — Supplementary material 1. [file 40644_2024_692_MOESM1_ESM.docx]

**SUPPLEMENTARY DATA**

1. **The equipment and procedure of CT-guided percutaneous RFA**

One multi-needle extension unipolar RF system (MedSphere International) with the mode of temperature control or impedance control for choice. The power settings were adjusted according to the manufacturer’s protocols: 5 min for a 2.0 ~ 2.5 cm active tip at 30 W, 8 min for a 3.0 ~ 3.7 cm active tip at 50 W, and 10 min for a 4.0 ~ 4.7 cm active tip at 60 W, respectively. Another commercially available electrode **system** with generators (Cool-tip RF System**,** Olympus) and an internally cooled, 17-gauge, 15 cm, electrode, with a 2 or 3 cm long ‎exposed ‎metallic tip was used.

The RFA procedure was performed under CT guidance ‎by three senior interventional radiologists with over 10 years of experience. Depending on the location of the target nodules, patients were placed in a prone position, lateral position, or supine position to ensure the best puncture site and entry route and avoid important structures, including ribs, interlobular fissures, and blood vessels. Lidocaine (2%, 5ml) was administered at the puncture site to induce local anesthesia of the pleura. With CT monitoring, the radiofrequency antennas were inserted through a single pleural puncture according to the predetermined direction and angle. The ablation was not performed until the CT scan confirmed that the electrode hooked the lesion. Considering the tumor shape and size, the energy output was adjusted to achieve complete ablation until the entire tumor was completely covered by ground-glass opacity (GGO; indicating the ablation area) according to the manufacturer’s protocols. The RFA course was completed by needle-path cauterization under thermal coagulation to avoid tumor cell implantation. A repeat CT (same parameters) scan was performed to evaluate whether the ablation zones covered the tumor and the occurrence of ablation-related complications, mainly including pneumothorax and hemorrhage. Patients were required to remain on bed rest for the first 8-hour after RFA, during which they were closely monitored by physicians.

1. Radiomics features employed in this study

The radiomics features employed in this study included histogram features, shape features, grey level co-occurrence matrix-based features (GLCM Features), grey level run length matrix-based features (GLRLM Features), grey level size zone matrix-based features (GLSZM Features), neighboring grey tone difference matrix-based features (NGTDM Features) and wavelet-transform based features (Wavelet Features), which were listed in Table S1.

| **Histogram Features** | **NGTDM Features** | **Wavelet Features** | | **Shape Features** | |
| --- | --- | --- | --- | --- | --- |
| Variance | Coarseness | HHH Category | LLL Category | Tumor Volume | Compactness 1 |
| Skewness | Contrast | HLH Category | LHH Category | Surface Area | Compactness 2 |
| Kurtosis | Busyness | HLL Category | LHL Category | Surface Area to Volume ratio | Spherical Disproportion |
| Uniformity | Complexity | HHL Category | LLH Category | Sphericity | Flatness |
| Energy | Strength |  |  |  |  |
| Entropy |  |  |  |  |  |
| **GLCM Features** | | **GLRLM Features** | | **GLSZM Features** | |
| Autocorrelation | Cluster Shade | Short Run Emphasis | | Emphasis | Zone Percentage |
| Contrast | Sum entropy | Long Run Emphasis | | Small Zone Emphasis | |
| Correlation 1 | Sum average | Grey-level Non-uniformity | | Large Zone Emphasis | |
| Correlation 2 | Sum variance | Run-Length Non-uniformity | | Grey-Level Non-uniformity | |
| Cluster Prominence | Sum of squares Variance | Run Percentage | | Zone-Size Non-uniformity | |
| Maximum probability | Difference variance | Low Grey-Level Run Emphasis | | Zone-Size Variance | |
| Dissimilarity | Difference entropy | High Grey-Level Run Emphasis | | Grey-Level Variance | |
| Energy | Homogeneity | Short Run Low Grey-Level Emphasis | | Low Grey-Level Zone | |
| Entropy | Homogeneity 2 | Short Run High Grey-Level Emphasis | | High Grey-Level Zone Emphasis | |
| Information measure of correlation 1 | | Long Run Low Grey-Level Emphasis | | Small Zone Low Grey-Level Emphasis | |
| Information measure of correlation 2 | | Long Run High Grey-Level Emphasis | | Small Zone High Grey-Level Emphasis | |
| Inverse difference normalized | | Grey-Level Variance | | Large Zone Low Grey-Level Emphasis | |
| Inverse difference moment normalized | | Run-Length Variance | | Large Zone High Grey-Level Emphasis | |

**Table S1.** **Radiomics features employed in this study.**

The ‘Wavelet Features’ are radiomics features which are extracted from the images pre-processed by wavelet-transform, as shown in Figure S1. For example, the HLH Category wavelet features were the radiomics features derived from images decomposed by high-pass wavelet transform on the X-axis, low-pass wavelet transforms on the Y-axis and high-pass wavelet transform on the Z-axis.

| **Firstorder** | **Glcm** | **Glrlm** | **Glszm** |
| --- | --- | --- | --- |
| 10Percentile | Autocorrelation | GrayLevelNonUniformity | GrayLevelNonUniformity |
| 90Percentile | ClusterProminence | GrayLevelNonUniformityNormalized | GrayLevelNonUniformityNormalized |
| Energy | ClusterShade | GrayLevelVariance | GrayLevelVariance |
| Entropy | ClusterTendency | HighGrayLevelRunEmphasis | HighGrayLevelZoneEmphasis |
| InterquartileRange | Contrast | LongRunEmphasis | LargeAreaEmphasis |
| Kurtosis | Correlation | LongRunHighGrayLevelEmphasis | LargeAreaHighGrayLevelEmphasis |
| Maximum | DifferenceAverage | LongRunLowGrayLevelEmphasis | LargeAreaLowGrayLevelEmphasis |
| Mean | DifferenceEntropy | LowGrayLevelRunEmphasis | LowGrayLevelZoneEmphasis |
| MeanAbsoluteDeviation | DifferenceVariance | RunEntropy | SizeZoneNonUniformity |
| Median | Id | RunLengthNonUniformity | SizeZoneNonUniformityNormalized |
| Minimum | Idm | RunLengthNonUniformityNormalized | SmallAreaEmphasis |
| Range | Idmn | RunPercentage | SmallAreaHighGrayLevelEmphasis |
| RobustMeanAbsoluteDeviation | Idn | RunVariance | SmallAreaLowGrayLevelEmphasis |
| RootMeanSquared | Imc1 | ShortRunEmphasis | ZoneEntropy |
| Skewness | Imc2 | ShortRunHighGrayLevelEmphasis | ZonePercentage |
| TotalEnergy | InverseVariance | ShortRunLowGrayLevelEmphasis | ZoneVariance |
| Uniformity | JointAverage |  | **Ngtdm** |
| Variance | JointEnergy |  | Busyness |
|  | JointEntropy |  | Coarseness |
|  | MaximumProbability |  | Complexity |
|  | SumEntropy |  | Contrast |
|  | SumSquares |  | Strength |

Fig. S1 Schematic of the wavelet transform applied to CT images.

I: the original image; High: high-pass wavelet transforms; Low: low-pass wavelet transforms; $I_{HHH},I_{HHL},I_{HLH},I_{HLL},I_{LHH},I_{LHL},I_{LLH},I_{LLL}$: images decomposed by directional wavelet transform.

**Table S2. Radiomics features employed in the Habitat generation.**

**References:**

1. Haralick R M, Shanmugam K and Dinstein I 1973 Textural features for image classification IEEE Trans. Syst. Man Cybern. 3 610–21.

2. Galloway M M 1975 Texture analysis using gray level run lengths Comput. Graph. Image Process.4 172–9.

3. Thibault G, Fertil B, Navarro C, Pereira S, Cau P, Levy N, Sequeira J and Mari J-L 2009 Texture indexes and gray level size zone matrix: application to cell nuclei classification Pattern Recognition and Information Processing (PRIP) (Minsk, Belarus) pp 140–5.

4. Amadasun M R 1989 Textural features corresponding to textural properties IEEE Trans. Syst. Man Cybern. 19 1264–74.

5. Vallieres M 2015 A radiomics model from joint FDG-PET and MRI texture features for the prediction of lung metastases in soft-tissue sarcomas of the extremities. Phys. Med. Biol. 60 5471-5496.

1. Several commonly used machine learning models were utilized to construct models, including logistic regression (LR), support vector machine (SVM), K-nearest neighbor (KNN), random forest (RF), extremely randomized trees (ExtRa Trees), eXtreme gradient boosting (XGBoost), light gradient boosting machine (LightGBM), and multi-layer perceptron (MLP).


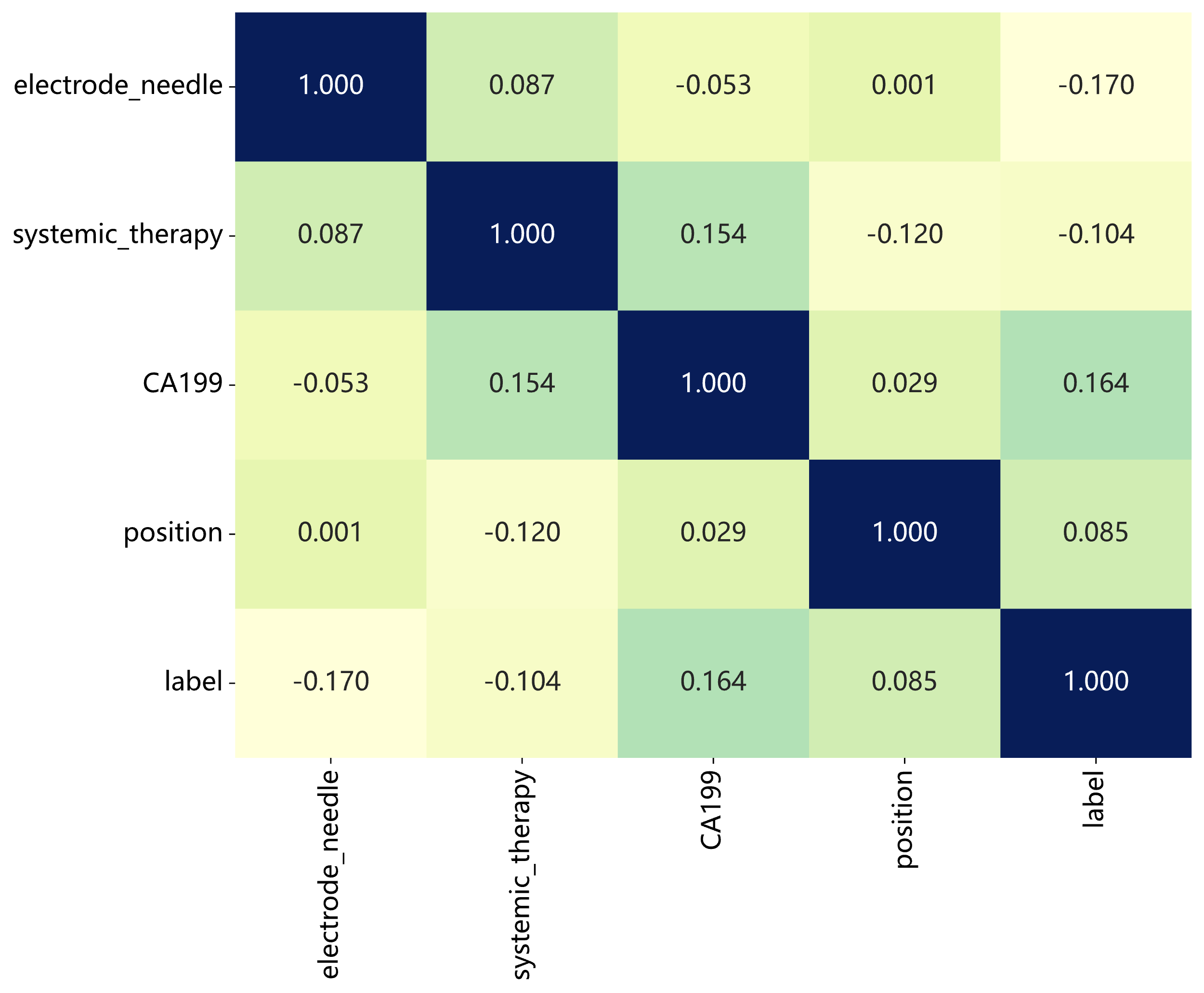


Fig. S2 The Spearman correlation coefficients of the clinical variables with P < 0.05 in the multivariable regression analysis.


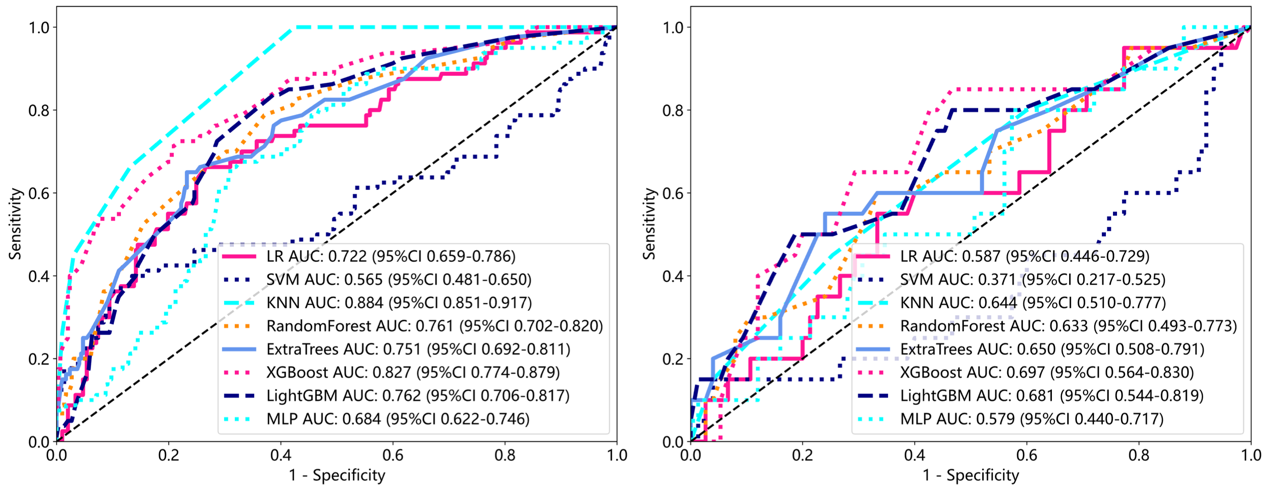


Fig. S3 The receiver operating characteristic (ROC) curves of clinical models based on different machine learning models in the training cohort (a), and test cohort (b). **XGBoost** was chosen to construct the **clinical** signature.


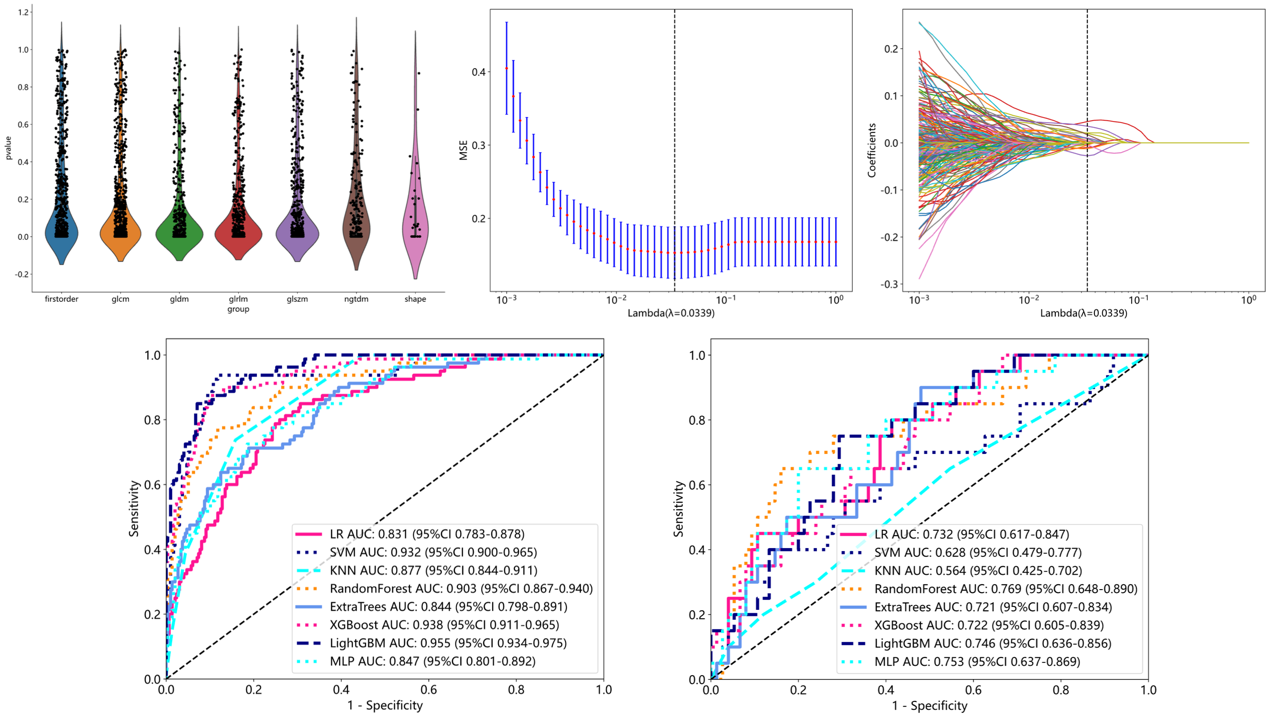


Fig. S4 The distribution of radiomics features (a), tuning parameter (λ) selection in the least absolute shrinkage and selection operator (LASSO) model used 10-fold cross-validation (b), LASSO coefficient profiles of radiomics features (c), the receiver operating characteristic (ROC) curves of Intra radiomics models based on different machine learning models in the training cohort (d), and test cohort (e). RandomForest was chosen to construct the Intra radiomics signature.


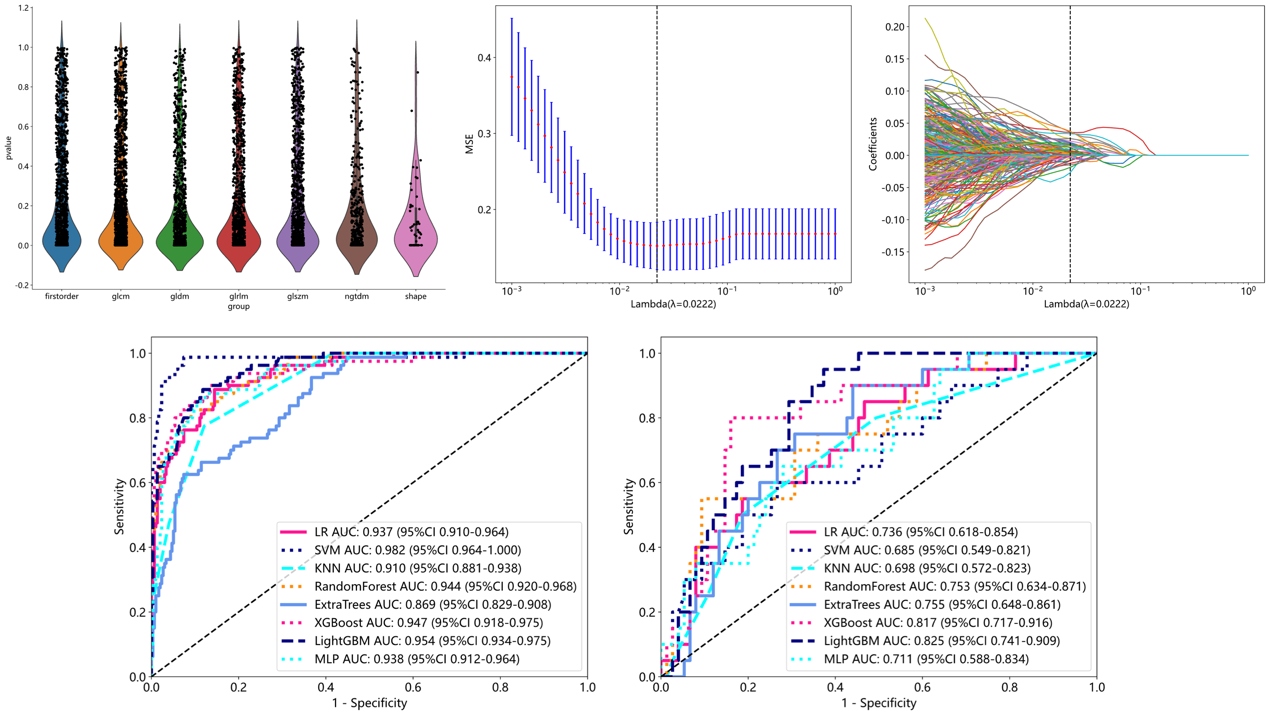


Fig. S5 The distribution of radiomics features (a), tuning parameter (λ) selection in the least absolute shrinkage and selection operator (LASSO) model used 10-fold cross-validation (b), LASSO coefficient profiles of radiomics features (c), the receiver operating characteristic (ROC) curves of **Peri-5** radiomics models based on different machine learning models in the training cohort (d), and test cohort (e). **LightGBM** was chosen to construct the Peri-5 radiomics signature.


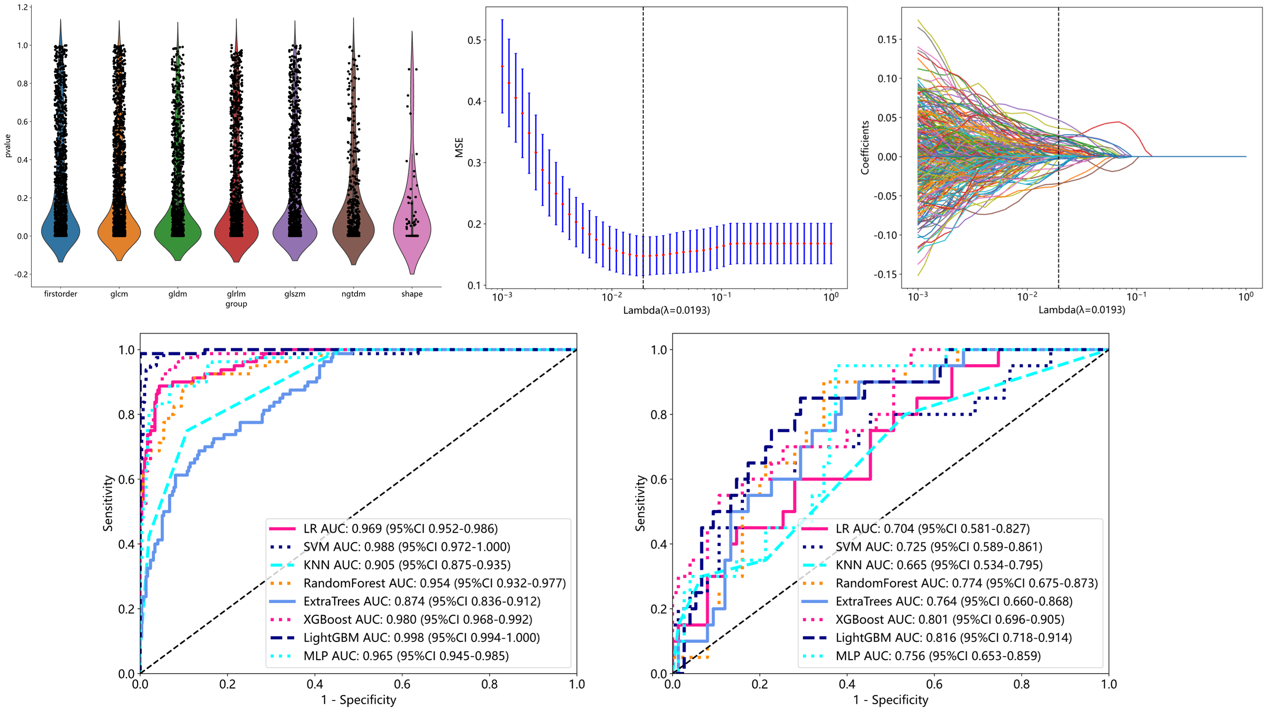


Fig. S6 The distribution of radiomics features (a), tuning parameter (λ) selection in the least absolute shrinkage and selection operator (LASSO) model used 10-fold cross-validation (b), LASSO coefficient profiles of radiomics features (c), the receiver operating characteristic (ROC) curves of **Peri-10** radiomics models based on different machine learning models in the training cohort (d), and test cohort (e). **LightGBM** was chosen to construct the Peri-10 radiomics signature.


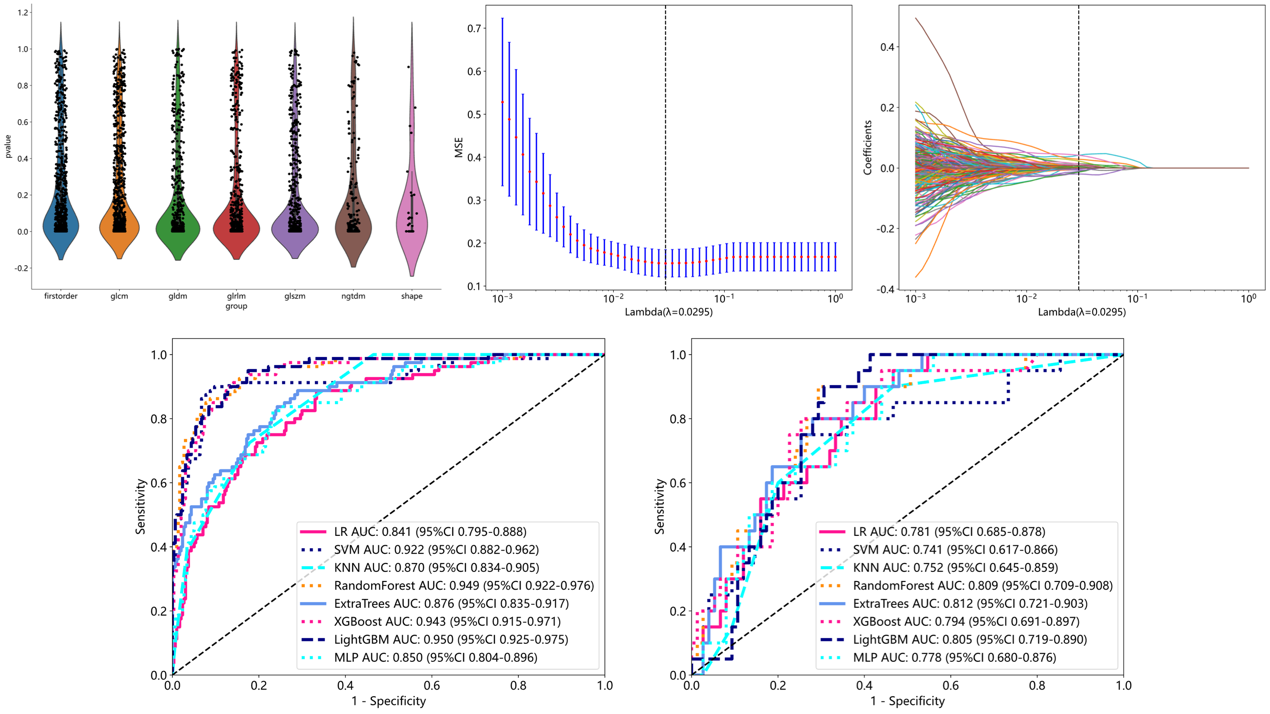


Fig. S7 The distribution of radiomics features (a), tuning parameter (λ) selection in the least absolute shrinkage and selection operator (LASSO) model used 10-fold cross-validation (b), LASSO coefficient profiles of radiomics features (c), the receiver operating characteristic (ROC) curves of **Habitat** radiomics models based on different machine learning models in the training cohort (d), and test cohort (e). **ExtRaTrees** was chosen to construct the Habitat radiomics signature.


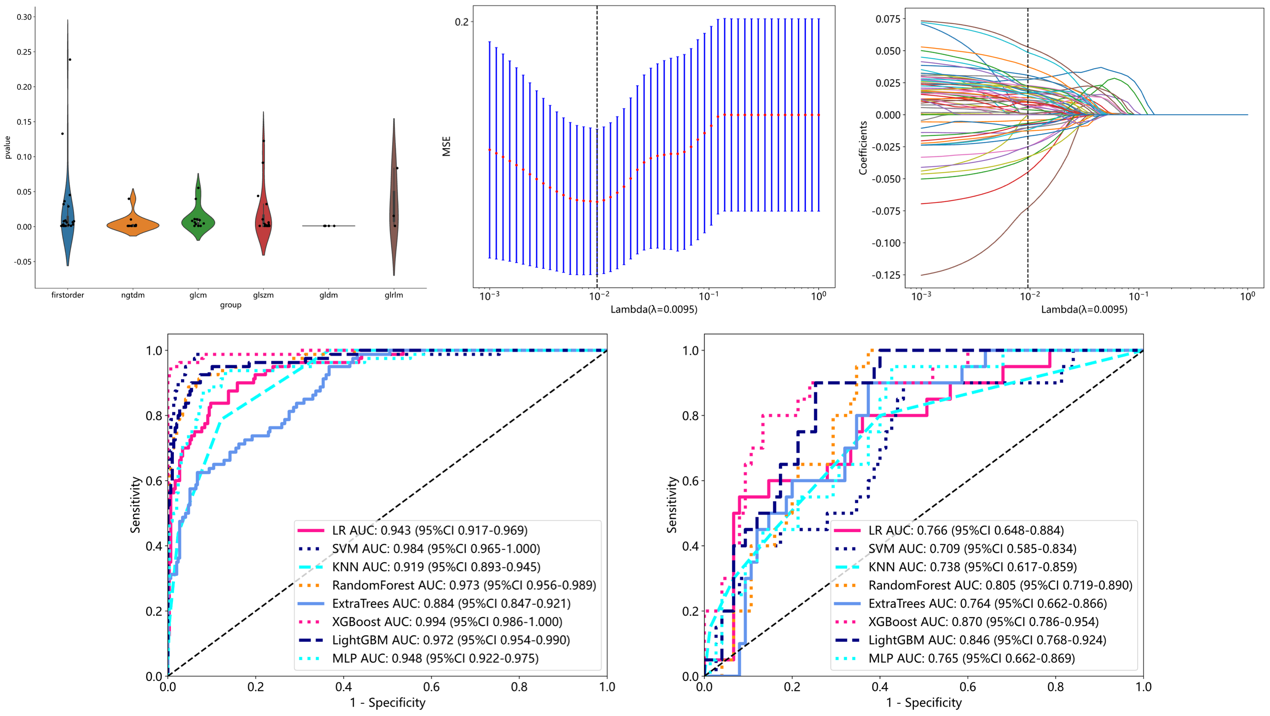


Fig. S8 The distribution of radiomics features (a), tuning parameter (λ) selection in the least absolute shrinkage and selection operator (LASSO) model used 10-fold cross-validation (b), LASSO coefficient profiles of radiomics features (c), the receiver operating characteristic (ROC) curves of **Habitat+Peri-5** radiomics models based on different machine learning models in the training cohort (d), and test cohort (e). **XGBoost** was chosen to construct the Habitat+Peri-5 radiomics signature.
